# Applications of the Photocatalytic Degradation of TiO_2_ Nanoparticles Under UV Radiation in the Development of Innovative Self-Cleaning Geopolymer Construction Materials

**DOI:** 10.3390/polym18060697

**Published:** 2026-03-12

**Authors:** Andreea Hegyi, Adrian-Victor Lăzărescu, Tudor Panfil Toader, Carmen Florean

**Affiliations:** National Institute for Research & Development URBAN-INCERC Cluj-Napoca Branch, 117 Calea Florești, 400524 Cluj-Napoca, Romania; andreea.hegyi@incerc-cluj.ro (A.H.); tudor.toader@incerc-cluj.ro (T.P.T.); carmen.florean@incerc-cluj.ro (C.F.)

**Keywords:** geopolymer, alkaline activation, self-cleaning capacity, TiO_2_ nanoparticles

## Abstract

Geopolymer materials obtained through the alkaline activation of fly ash represent a promising alternative for reducing the environmental impact of the construction sector, which is currently dominated by cement use. This study aimed to develop self-cleaning geopolymer composites by incorporating TiO_2_ nanoparticles. Specimens containing 1%, 3%, and 4% TiO_2_ were prepared using alkaline solutions based on Na_2_SiO_3_ and NaOH (6 M or 8 M), at mass ratios of 1:1 and 2:1. The results indicate that the three analyzed factors—the NaOH solution concentration, the activator ratio, and the nanoparticle dosage—significantly influence density, mechanical strength, and water absorption. Increasing the NaOH concentration to 8 M led to slight densification, improved flexural and compressive strength, and reduced water absorption. Modifying the Na_2_SiO_3_:NaOH ratio produced similar densification effects but resulted in reductions in mechanical strengths. The addition of 1–3% TiO_2_ increased density and mechanical performance while reducing water absorption, whereas 4% TiO_2_ content had the opposite effect. Self-cleaning capacity was confirmed by up to ~90% degradation of Rhodamine B after five UV–artificial rain–drying cycles, compared to only 27.3% degradation for the control samples.

## 1. Introduction

It is well known that cement, as the main raw material, and cement-based composites, as derived materials, currently represent a rapid and technologically well-established solution for the construction of long-lasting buildings with diverse functions, ranging from residential use to public buildings, industrial facilities, bridges, parapets, and others. However, cement production technology is highly polluting and energy-intensive, generating significant environmental concerns. The Portland cement industry is responsible for approximately 36% of total energy consumption and 39% of CO_2_ emissions within the overall construction industry [1].

Therefore, at a global level, one of the solutions identified for reducing the carbon footprint of construction, while simultaneously reintegrating an industrial by-product into the economic circuit—namely fly ash—has been the development of alkali-activated fly ash-based geopolymer binders [2,3,4,5].

At the same time, due to the rapid increase in the surface area and number of constructions, the need for maintenance and preservation of their appearance and hygiene introduces another environmental challenge: high water consumption and extensive use of cleaning and finishing restoration materials for both interior and exterior walls, along with the generation of significant amounts of dust and other pollutants during maintenance works. A potential solution to mitigate this issue could be the development of materials with enhanced resistance to soiling and self-cleaning capacity [1,6].

In this context, the exploitation of nanomaterials and nanotechnologies represents one of the promising approaches. Specialized research has shown that ZnO or TiO_2_ nanoparticles incorporated initially into cementitious composites and later into geopolymers contribute to improved performance and, under UV radiation, enable the development of self-cleaning properties [6,7,8,9,10,11,12,13,14].

In the case of TiO_2_ nanoparticles, the mechanism responsible for inducing self-cleaning capacity is based on the specific effect of UV radiation energy, which promotes an electronic transition at the valence band level. Titanium dioxide, being a semiconductor with a band gap of approximately 3.0 eV, generates electrons (e^−^) and holes (h^+^) upon energy absorption. The electrons tend to reduce Ti(IV) cations to Ti(III), while the holes oxidize O^2−^ anions. This process releases oxygen and creates vacancies on the TiO_2_ surface, allowing water molecules to bind and generate hydroxyl groups (OH^−^).

The photogenerated holes (h^+^) increase bond lengths within the TiO_2_ lattice, bringing the surface into a metastable state that permits molecular water adsorption, along with the formation of new hydroxyl groups and proton release. These hydroxyl groups are thermodynamically less stable; therefore, the surface promotes spreading of water droplets over a larger area to achieve stabilization [15,16,17,18,19]. This results in superhydrophilicity, meaning that water reaching the photoactivated composite surface no longer forms discrete droplets but spreads as a thin film that facilitates the removal of impurities. The induction of hydrophilicity represents the first key element in achieving self-cleaning capacity.

Simultaneously, under UV radiation, electron–hole pairs (e^−^/h^+^) react with O_2_ and H_2_O, generating reactive oxygen species such as superoxide anion radicals (O_2_•^−^) and hydroxyl radicals (•OH). These oxidative species (h^+^, O_2_•^−^, and •OH) are highly reactive and initiate and propagate oxidation–reduction reactions of the organic matter constituting surface stains. Initially large and complex organic molecules are progressively transformed into simpler, smaller compounds until complete degradation occurs, forming H_2_O and CO_2_ [20]. These oxidation–reduction reactions, together with superhydrophilicity, constitute the second essential component of the self-cleaning mechanism.

In addition to self-cleaning capacity, the existing literature indicates that incorporating TiO_2_ nanoparticles (NPs) into geopolymer matrices also induces microstructural modifications, leading to variations in physico-mechanical properties. Several studies have demonstrated that NP addition improves mechanical strength, carbonation resistance, and material densification, reducing microcracking risk by promoting geopolymerization reactions [21,22]. Guerrero et al. [23] attribute these improvements also to the microfiller effect of NT, which fills pores and modifies their volume and distribution.

However, significant controversy remains regarding the optimal NP dosage. Duan et al. [21] report an optimal content of 5 wt.% NPs for improving physico-mechanical performance, while Sastry et al. [24] suggest a favorable range between 2.5 and 5 wt.%. Guerrero et al. [23] indicate 1 wt.% NPs as optimal for maximizing self-cleaning efficiency in Rhodamine B tests, whereas Yang et al. [22] report an optimal requirement of 10 wt.% NT.

Based on the above considerations, the objective of this study is to analyze the influence of TiO_2_ nanoparticle incorporation on the physico-mechanical properties and on the potential to induce a smart, innovative self-cleaning behavior of geopolymer matrices prepared using locally available raw materials.

## 2. Materials and Methods

The experimental program was designed to investigate two main hypotheses:

**Hypothesis 1.** 
*The addition of TiO_2_ nanoparticles (NPs) to the geopolymer matrix leads to changes in the physico-mechanical parameters of the hardened composites.*

**Hypothesis 2.** 
*Upon photoactivation under UV radiation (incident wavelength < 400 nm), the TiO_2_ nanoparticles incorporated in the geopolymer composite—present as a mixture of anatase and rutile crystalline phases—become activated and impart a self-cleaning property to the geopolymer surface.*

Based on these hypotheses, both direct, measurable quantitative indicators and qualitative indicators were identified. Thus, the influence of NP addition on the physico-mechanical characteristics of the composite was quantified using the following quantitative indicators:

- Apparent density in hardened state/variation in apparent density compared to the control sample (without NP content);
- Flexural tensile strength/variation in flexural tensile strength compared to the control sample (without NP content);
- Compressive strength/variation in compressive strength compared to the control sample (without NP content);
- Water absorption coefficient by capillarity/variation in the water absorption coefficient by capillarity compared to the control sample (without NP content).

For the analysis of self-cleaning capacity, the following qualitative indicators were used:

- Surface whiteness degree, measuring the surface whiteness after UV photoactivation, after staining, and after completing photoactivation–washing–drying cycles;
- Degree of recovery of surface whiteness after completing photoactivation–washing–drying cycles.

**Figure 1:**
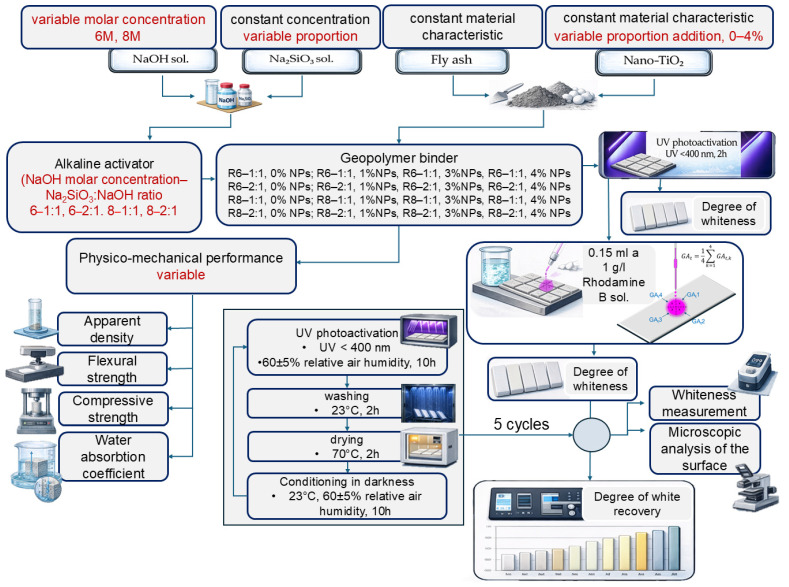
Schematic representation of the experimental methodology.

The methodology for preparing the geopolymer mixtures, manufacturing the specimens, and performing the experimental testing is schematically presented in Figure 1.

### 2.1. Raw Materials, Preparation of Geopolymer Composites, and Specimen Fabrication

**Figure 2:**
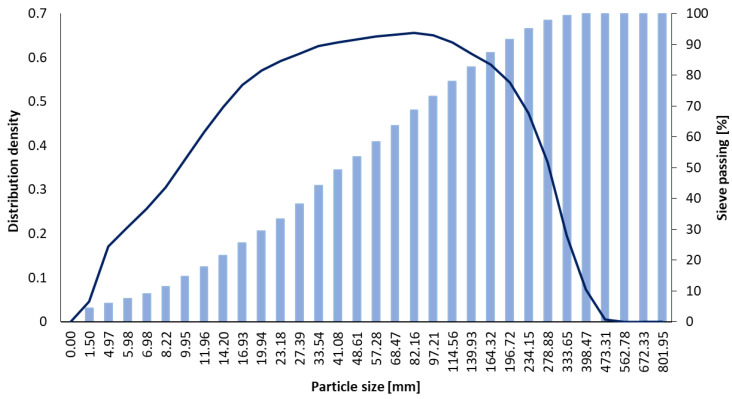
Distribution density and sieve passing of the fly ash used in the production of the samples.

For the experimental investigations, the raw materials used for preparing the geopolymer binder included fly ash (FA) sourced from the Rovinari thermal power plant (Romania), characterized in terms of mineralogical composition, oxide composition, and particle size distribution, as presented in Table 1 and Figure 2, along with an alkaline activator.

The physico-chemical characterization of the fly ash was performed using X-ray fluorescence (XRF) analysis with a HELOS RODOS/L, R5 instrument (Sympatec GmbH, Clausthal-Zellerfeld, Germany) (Table 1). The mineralogical composition of the fly ash was investigated using X-ray diffraction (XRD) analysis with a Bruker D8 ADVANCE X-ray diffractometer (Bruker, Karlsruhe, Germany). Scans were collected over a 2θ range of 5–60°, with a step size of 0.02° and a scanning speed of 10 s per step.

The preparation of the alkaline activator (AA) was carried out by mixing an aqueous NaOH solution with a Na_2_SiO_3_ solution. The NaOH aqueous solution was prepared by dissolving 99% purity NaOH pellets in distilled water at room temperature (23 ± 1 °C).

The Na_2_SiO_3_ solution was commercially purchased (Polichim, Baia Mare, Romania), characterized by a composition of 30% SiO_2_, 14% Na_2_O, and 56% H_2_O. The mass mixing ratios of the two solutions for obtaining the alkaline activator were Na_2_SiO_3_/NaOH = 1:1 and 2:1. Based on preliminary studies [25,26,27], the 1:2 mass ratio was not used due to the risk of efflorescence formation on the surface of the hardened geopolymer, caused by excess NaOH.

Fly ash activation was carried out using an alkaline activator/fly ash mass ratio of 0.92–0.95, established based on preliminary tests aimed at determining a ratio that would ensure the consistency parameter of the fresh composite fell within the spread diameter limits of 175 ± 10 mm, measured using the flow table method [28], for both control compositions and compositions containing TiO_2_ nanoparticles (NPs).

This slight adjustment of the alkaline activator/fly ash ratio was necessary considering that NPs induce increased water demand—i.e., alkaline activator demand in this case [25,26]. In other words, a slight increase in the amount of alkaline activator was required as the NP content increased, to provide sufficient liquid for proper activation of the constant fly ash content and to obtain a mixture with adequate workability, which is essential for maintaining subsequent physico-mechanical properties.

For each mixture, several variants were prepared, in which the control sample was defined as the composition without nanoparticle addition, while the experimental samples were produced by incorporating 1%, 3%, or 4% NPs, expressed as mass percentages relative to the fly ash content.

To produce the samples, AEROXIDE^®^ TiO_2_ P25 nanoparticles (Evonik Degussa Industries AG, Hanau, Germany) were used, characterized by an average particle size of 21 nm, a specific surface area of 35–65 m^2^/g, a purity of 99.5%, and containing at least 70% anatase crystalline phase.

The incorporation of NPs into the geopolymer matrix was performed by dry mixing the nanoparticles with the fly ash prior to alkaline activation.

Based on the above, the compositional characteristics and the sample coding system are presented in Table 2.

The preparation of the geopolymer mixtures followed a series of gravimetric pre-dosing steps for the raw materials using a KERN FKB 36K0.1 balance (KERN & SOHN GmbH, Balingen, Germany) with an accuracy of 0.1 g.

Subsequently, the NaOH solutions were prepared by dissolving the solid NaOH pellets in water in the quantitatively determined amounts required to obtain the two concentration variants, 6 M and 8 M. The liquid precursors of the alkaline activator (NaOH solution of the specified concentration and Na_2_SiO_3_ solution used as received) were manually mixed, and the resulting solution was stored under laboratory conditions (23 ± 1 °C and 65 ± 5% relative humidity) in a sealed container until use.

The powdered materials, fly ash (FA) and TiO_2_ nanoparticles (NPs), were dry-mixed using an ELLE paddle mixer (ELLE International Ltd., Milton Keynes, UK) at low speed and in a covered container to prevent material loss. The pre-dosed amount of alkaline activator was then added to the dry mixture, and homogenization was carried out using the same ELLE paddle mixer.

From each mixture, a sample was evaluated in terms of workability. For mixtures with NT, the amount of alkaline activator (AA) was slightly adjusted in controlled increments until the target spread diameter of 175 ± 10 mm was achieved using the flow table method [28].

After preparation, prismatic specimens measuring 40 × 40 × 160 mm were cast for physico-mechanical testing, and prismatic specimens measuring 15 × 65 × 85 mm were prepared for self-cleaning capacity assessment. Casting was performed using metal molds and plastic molds. After casting, all specimens were conditioned for 24 h at 70 ± 2 °C in a thermostatic chamber (MEMMERT ULE 500, MEMMERT GmbH + Co., KG, Schwabach, Germany) to ensure the completion of geopolymerization reactions. The specimens were then demolded and cured for an additional 6 days under laboratory conditions (23 ± 1 °C, 65 ± 5% relative humidity, and without light exposure).

### 2.2. Analysis of Physico-Mechanical Performance

After curing, the geopolymer composites were characterized in terms of their physico-mechanical and structural properties. Following testing standards specific to mortars, the apparent density in the hardened state (EN 1015-10:2002+A1:2007) [29], flexural tensile strength and compressive strength (EN 1015-11:2020) [30], and water absorption by capillarity (EN 1015-18:2003) [31] were determined using prismatic specimens of 40 × 40 × 160 mm. The dimensional characterization of the specimens was carried out by direct measurement using a digital electronic caliper (INSIZE Czech s.r.o., Ivančice, Czech Republic). Weighing was performed using a KERN FKB 36K0.1 balance (KERN & SOHN GmbH, Balingen, Germany) with an accuracy of 0.1 g, and mechanical strengths were determined using an ELE-type testing machine (ELE International Ltd., Milton Keynes, UK). The number of tested specimens complied with the specifications of the testing standards to ensure repeatability and reproducibility, and the results were reported as the arithmetic mean of the individual values.

### 2.3. Evaluation of Self-Cleaning Performance

For each type of geopolymer composition, a batch of three specimens with dimensions of 15 × 65 × 85 mm, cured to maturity (7 days after casting), was analyzed by recording the surface whiteness degree using a WBS-1 type leucometer, under laboratory conditions, with a constant illumination level and constant light incidence on the analyzed surface. The whiteness degree is defined on a scale from 0 to 100 measuring units (MU), where 0 MU corresponds to absolute black and 100 MU to absolute white.

Subsequently, the specimens were subjected to photoactivation by exposure for 2 h to UV radiation emitted in the 320–400 nm range, corresponding to the UVA band (which represents about 95% of the UV radiation component of sunlight reaching the Earth’s surface) [32,33,34]. Moreover, since TiO_2_ (especially the anatase phase) has a band gap of approximately 3.2 eV, it can only be activated by photons with wavelengths λ ≤ 387 nm, thereby triggering the redox processes required to induce the self-cleaning properties of the material [10,34]. The UVA radiation source was positioned at 10 cm above the specimen surface, resulting in a luminous flux intensity of 860 lux, after which the surface whiteness degree was recorded again.

For each batch of specimens, the whiteness degree was determined again, and the variation in whiteness degree after UV photoactivation (VGA_UV_) was calculated as the percentage increase in specimen whiteness after UV photoactivation relative to the initial whiteness, as shown in Equation (1).

(1) $${VGA}_{UV}=\frac{{{GA}_{UV}-GA}_{i}}{{GA}_{i}}\times 100\;(\%)$$

where GA_i_—initial whiteness degree; and GA_UV_—whiteness degree after UV photoactivation.

Subsequently, 0.15 mL of a Rhodamine B solution with a concentration of 1 g/L was dropwise applied onto the surface of the specimens. The surface whiteness degree in the stained area was measured again as the average of four individual readings, and the percentage reduction in whiteness due to staining (SGA_Rh_) was calculated relative to the whiteness degree of the unstained, UV-photoactivated specimen (Equation (2)).

(2) $${SGA}_{Rh}=\frac{{{GA}_{UV}-GA}_{Rh}}{{GA}_{UV}}\times 100\;(\%),$$

where GA_UV_—whiteness degree after initial UV photoactivation; and GA_Rh_—whiteness degree immediately after staining with Rhodamine B solution.

After staining, the specimens were subjected to a testing cycle consisting of a sequence of exposures to UV radiation (10 h, 860 lux)–artificial rain washing (2 h)–drying (2 h, 70 °C)–storage in dark conditions (10 h, temperature 23 ± 1 °C, relative air humidity 65 ± 5%), in order to simulate day–night cycles with periods of sunlight and rainfall. During the artificial rain exposure, the specimens were placed on a support allowing a 30% inclination angle relative to the horizontal to facilitate water runoff from the surface, while during the other stages, the specimens were positioned horizontally, which allowed perpendicular incidence of UV rays on the geopolymer surface during the photoactivation stage.

At the end of each of the five UV radiation (10 h, 860 lux)–artificial rain washing (2 h)–drying (2 h, 70 °C)–dark storage (10 h, 23 ± 1 °C, 65 ± 5% relative humidity) cycles, the surface whiteness degree in the stained area was measured for each specimen. Using the mean whiteness degree values obtained after a given number of photoactivation–washing–drying cycles and normalizing them to the whiteness degree recorded after photoactivation of the specimens prior to staining, the whiteness recovery degree (RGA_i_) was calculated as a quantitative indicator of the surface’s self-cleaning capacity (Equation (3)).

(3) $${RGA}_{i}=\frac{{GA}_{i}}{{GA}_{UV}}\times 100\;(\%),$$

where GA_UV_—whiteness degree after initial UV photoactivation; GA_i_—whiteness degree measured after completing i full cycles of UV radiation (10 h, 860 lux)–artificial rain washing (2 h)–drying (2 h, 70 °C)–dark storage (10 h, 23 ± 1 °C, 65 ± 5% relative humidity); and i = 1–5 cycles.

At the end of the five complete cycles of UV radiation (10 h, 860 lux)–artificial rain washing (2 h)–drying (2 h, 70 °C)–dark storage (10 h, 23 ± 1 °C, 65 ± 5% relative humidity), the self-cleaning efficiency indicator (η) was calculated (Equation (4)). This parameter represents the percentage ratio between the amount of whiteness recovered after cleaning and the amount lost due to staining; in other words, the percentage of the initial loss that was recovered.

(4) $$\eta =\frac{{{GA}_{5}-GA}_{Rh}}{{GA}_{UV}-{GA}_{Rh}}\times 100\;(\%),$$

where GA_UV_—whiteness degree after initial UV photoactivation; GA_Rh_—whiteness degree immediately after staining with Rhodamine B solution; and GA_5_—whiteness degree after completing the five full cycles of UV radiation (10 h, 860 lux)–artificial rain washing (2 h)–drying (2 h, 70 °C)–dark storage (10 h, 23 ± 1 °C, 65 ± 5% relative humidity).

In addition, a visual analysis of the specimen’s surface appearance was performed by microscopically examining the stained area using a LEICA SAPO stereomicroscope (Leica Microsystems GmbH, Wetzlar, Germany).

Each time the whiteness degree was measured, for each specimen, individual readings were taken at four points located in the central area of the specimen, in the four quadrants of a circle with a constant diameter (20 mm) defined by two perpendicular diameters. The indicator value was reported as the arithmetic mean of the four individual readings. This methodology for defining the measurement points of the whiteness degree was maintained throughout all determinations, including after staining and after exposure to photoactivation–washing–drying cycles, with the staining agent being applied within the delimited working area in order to increase the accuracy of the measurements.

## 3. Results and Discussions

**Figure 3:**
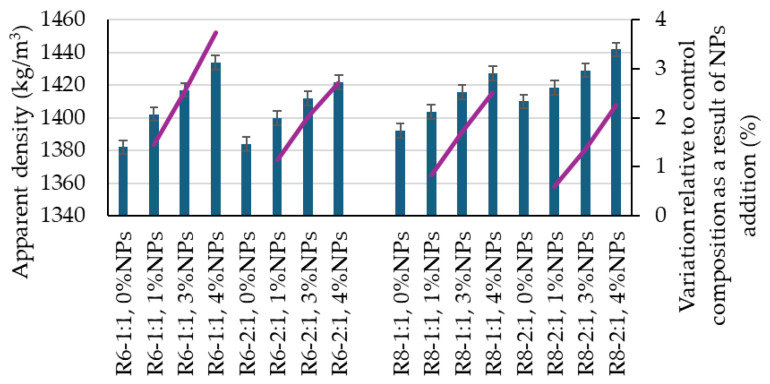
Influence of mix design ratio on the density of geopolymer composites.

From the perspective of physico-mechanical characteristics, the reference geopolymer samples exhibit apparent density values in hardened state within the range of 1382–1410 kg/m^3^ (Figure 3). These values are influenced by the compositional design, namely the molarity of the NaOH solution and the Na_2_SiO_3_/NaOH ratio, which are characteristic of the alkaline activator used for the geopolymerization of fly ash. In this respect, an increase in the Na_2_SiO_3_:NaOH ratio leads to a slight densification tendency of the material.

**Figure 4:**
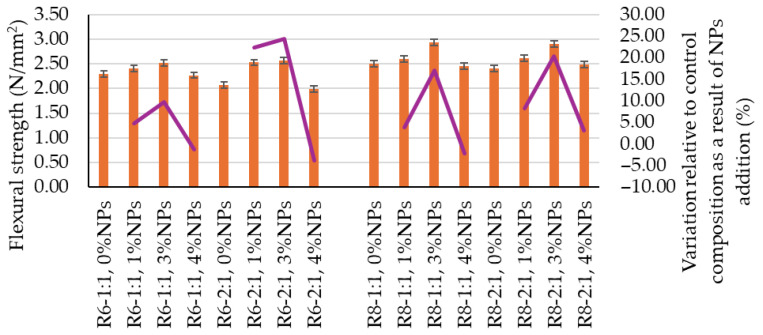
Influence of mix design ratio on the flexural tensile strength of geopolymer composites.

**Figure 5:**
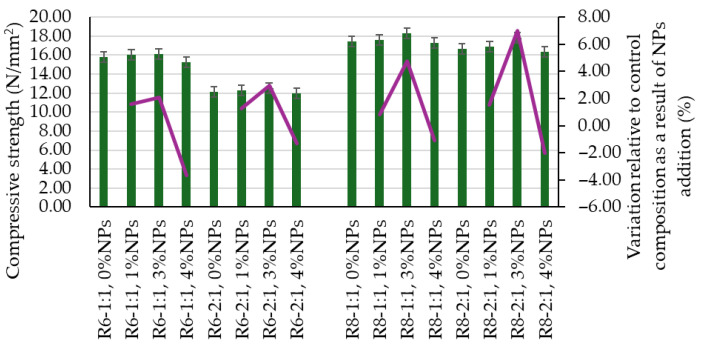
Influence of mix design ratio on the compressive strength of geopolymer composites.

Similarly, the influence of these two factors is also reflected in the mechanical strengths (Figure 4 and Figure 5); however, in this case, an increase in the Na_2_SiO_3_:NaOH ratio results in a slight decrease in performance. An increase in the molarity of the NaOH solution has a beneficial effect, as observed in Figure 3, Figure 4 and Figure 5, since mixtures prepared with 8 M NaOH solution are slightly denser and exhibit slightly higher mechanical strengths compared to the corresponding mixtures prepared with an alkaline activator obtained using a 6 M NaOH solution.

**Figure 6:**
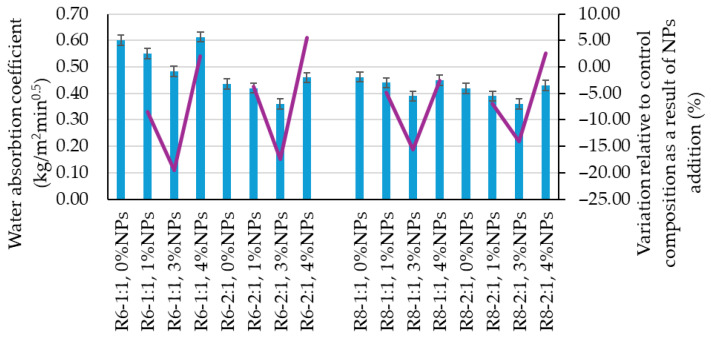
Influence of mix design ratio on the capillary water absorption coefficient of geopolymer composites.

In terms of the water absorption coefficient by capillarity specific to the reference commixtures (Figure 6), the values fall within the range of 0.42–0.60 kg/m^2^·min^0.5^. A higher Na_2_SiO_3_/NaOH ratio leads to a reduction in this indicator, while a higher molar concentration of the NaOH solution (8 M) also contributes to reducing water absorption by capillarity.

With the introduction of TiO_2_ nanoparticles, the characteristics of all geopolymer samples undergo changes. A progressive densification of the composite matrix is observed as the amount of added nanoparticles increases. Thus, for a 1 wt.% NP addition, an increase in the apparent density of the hardened composite of at least 0.59% (R8-1:2, 1% NT) and up to 1.46% (R6-1:1, 1% NT) is recorded compared to the reference mixtures (0% NT). In the case of a 3 wt.% nanoparticle addition, the increases are 1.36% (R8-1:2, 3% NT) and up to 2.54% (R6-1:1, 3% NT), while for a 4 wt.% nanoparticle addition, the increases are 2.26% (R8-1:2, 4% NT) and up to 3.74% (R6-1:1, 4% NT), relative to the apparent density of the reference compositions.

In all cases, it can be stated that mixtures prepared with an alkaline activator obtained using an 8 M NaOH solution exhibit smaller variations in apparent density in the hardened state as a result of TiO_2_ nanoparticle addition. Moreover, increasing the Na_2_SiO_3_:NaOH ratio (2:1) also leads to a reduction in the density variation in nanoparticle-containing composites compared to the corresponding reference mixtures.

The flexural tensile strength and compressive strength of geopolymer composites containing NPs are also influenced by the addition of nanoparticles to the geopolymer matrix. In this case, a general trend of improvement in these parameters can be identified up to a threshold level, beyond which further nanoparticle addition leads to an opposite effect, namely a reduction in mechanical strength. As shown in Figure 3 and Figure 4, both flexural tensile strength and compressive strength are enhanced for NP additions of 1 wt.% and 3 wt.%, with the recorded values being higher than those of the reference compositions. In the case of a 4 wt.% NP addition, these parameters decrease, except for the R8-2:1, 4% NP mixture, for which the flexural tensile strength is still higher than that of the corresponding reference sample (R8-2:1, 0 NT).

It can be observed that a 1 wt.% NP addition generally leads to modest increases in mechanical strength indicators. Thus, increases of up to 8.26% were recorded for flexural tensile strength, except for the R6-2:1, 1% NP mixture, which exhibited an increase of 22.41%. For compressive strength, increases below 2% were recorded for all analyzed cases. It is also noteworthy that a higher Na_2_SiO_3_:NaOH ratio (2:1) generally favors increases in mechanical strengths upon the addition of 1 wt.% NT.

Much more pronounced results are obtained for a 3 wt.% NP addition to the geopolymer matrices. In this case, the increase in flexural tensile strength ranges from a minimum of 9.71% (R6-1:1, 3% NT) to a maximum of 24.32% (R6-2:1, 3% NT), while the increase in compressive strength falls within the range of 2.06% (R6-1:1, 3% NT) to 6.98% (R8-2:1, 3% NT). However, no clear correlation could be established between the NaOH solution concentration used to prepare the alkaline activator and the magnitude of the variation induced by the nanoparticle addition.

The general tendency toward a reduction in mechanical strength indicators for 4 wt.% NP additions can be interpreted, in correlation with references from the specialized literature, as a sign of the limited ability of the geopolymer binder to homogeneously and uniformly incorporate nanoparticles. According to the specialized literature documenting such behavior [35,36,37,38], it is considered possible that, at higher contents, the nanoparticles may locally agglomerate, leading to an inhomogeneous distribution within the composite matrix and to the formation of local weak points due to the reduced amount of binder available to properly embed the nanoparticles.

Looking at these experimental results as a set of preliminary findings, this observation represents a hypothesis for the development of further, more in-depth studies aimed at analyzing geopolymer composite materials in greater detail, and the influence of the addition of nanoparticles (NPs), particularly from a microstructural perspective.

Overall, even though the flexural tensile strength of the R8-2:1, 4% NP composition does not decrease compared to the reference sample, a reduction in compressive strength is observed. Therefore, in this case as well, a 4 wt.% NP addition cannot be considered beneficial in terms of the mechanical performance of the composite. Consequently, from the perspective of physico-mechanical performance, a 3 wt.% TiO_2_ nanoparticle addition can be regarded as the upper effective limit. It should be noted, however, that since no experimental tests were performed for NP addition levels with smaller increments, the actual upper limit may be slightly higher, likely lying somewhere within the 3–4 wt.% range, without reaching 4 wt.%.

Capillary water absorption is also influenced by the addition of nanoparticles, as this parameter is known to be governed by the characteristic of open porosity. According to the specialized literature [39,40,41], the presence of NPs can induce changes in porosity in terms of pore volume, pore size, and pore size distribution within the composite matrix. As shown in Figure 6, NP additions of up to 3 wt.% lead to a reduction in the capillary water absorption coefficient, with decreases ranging from a minimum of 3.67% (R6-2:1, 1% NT) to a maximum of 19.58% (R6-1:1, 3% NT), which indirectly indicates a reduction in open porosity.

Considering the objective of this study, namely, to identify the possibility of inducing self-cleaning capabilities in geopolymer composites by exploiting the specific properties of nanoparticles (NPs), in correlation with the experimental results presented, new directions for continuing the research are identified as follows:

- Deepening the microstructural analysis of the modifications induced by the addition of NPs, by examining the influencing factors and correlating them with the specifications reported in the specialized literature, one of the objectives being to highlight, through specific testing methods (e.g., BET analysis), the changes induced at the level of porosity.
- Completing the experimental research by extending the compositional variants so that the amount of NPs is gradually increased using increments smaller than 1%, for example, initially using an incremental step of 0.25%, followed by a further reduction in the analysis interval and the increment step, in order to obtain a more detailed dataset, favorable for performing a rigorous statistical analysis and for identifying the optimal percentage of nanoparticle addition that leads to the desired material performance with greater precision. This approach, together with the use of specific microstructural analysis methods (XRD, SEM-EDS, BET, and FTIR), will allow a more accurate identification of the critical point, the underlying causes, and the optimal amount of NPs, so as to achieve an optimal balance between the macrostructural physical–mechanical characteristics and the self-cleaning capability of the composite.

In the case of a 4 wt.% NP addition, an increase in the capillary water absorption coefficient of up to 5.50% (R6-2:1, 4% NT) is generally recorded, except for the R8-1:1, 4% NP mixture. This behavior may further indicate an inhomogeneous distribution of nanoparticles within the geopolymer binder matrix. It can be observed that the largest reductions in capillary water absorption are obtained for the 3 wt.% NP addition, supporting the hypothesis that a 1 wt.% NP addition is beneficial but not optimal, while 4 wt.% NPs represents an unfavorable condition associated with nanoparticle excess.

For this indicator, no clear influence of the alkaline activator parameters—namely the Na_2_SiO_3_:NaOH ratio and the NaOH solution concentration—could be identified.

From the perspective of self-cleaning capacity, the experimental results are reflected by the evolution of the quantification indicators and by the comparative analysis of images illustrating the changes in surface appearance. From the outset, it should be noted that, since the staining agent is an aqueous solution, a potential influence of surface water absorption on the degree of staining and its subsequent evolution was anticipated, acting as an additional factor alongside those derived from the experimental design, namely the compositional formulation, nanoparticle addition, and the duration of exposure to photoactivation–washing–drying cycles.

**Figure 7:**
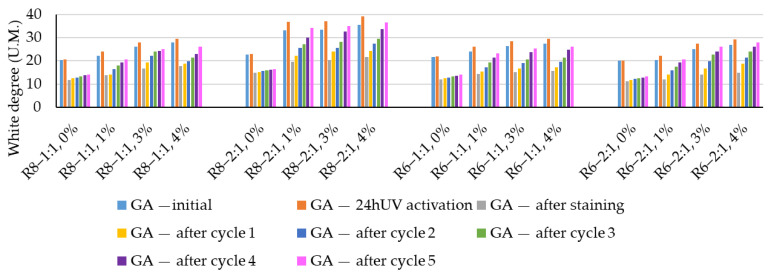
Degree of whiteness variation.

**Figure 8:**
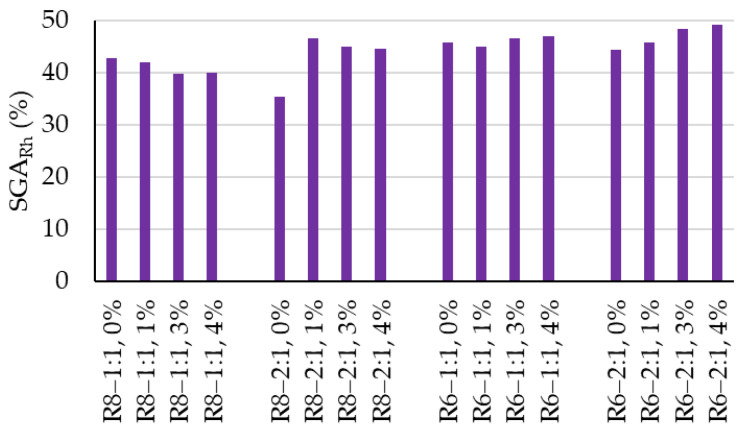
Percentage reduction in the degree of whiteness due to staining.

Figure 7 presents the mean values of the whiteness degree recorded for the analyzed cases and their evolution throughout the experimental program. After staining with the Rhodamine B solution, a drastic reduction in surface whiteness (SGA_Rh_) was observed (Figure 8), ranging from 39.8% to 49.2% relative to the whiteness degree of the unstained and UV-photoactivated surface. Although no clear correlations can be established with the geopolymer formulation, it is considered that the quantitative variation in the reduction in whiteness may be attributed to differences in surface and bulk porosity of the composites, which derive from the compositional design, while the other preparation parameters were kept constant.

**Figure 9:**
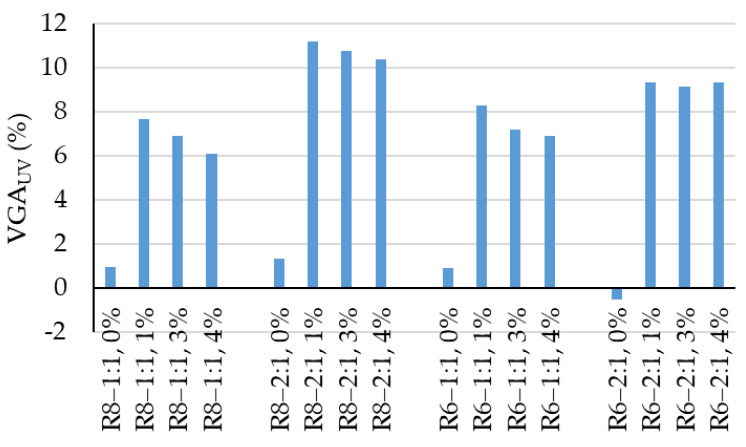
Variation in the degree of whiteness following UV photoactivation.

As shown in Figure 9, UV photoactivation leads to an increase in surface whiteness (VGA_UV_) of 6–11%. This improvement in whiteness exhibits a certain degree of non-uniformity depending on the compositional design of the geopolymer matrix as well as on the amount of NPs added. From this perspective, a 1 wt.% NP addition appears to be the most advantageous, as it yields the highest percentage increase in whiteness. As the NP content increases, the improvement in whiteness induced by UV photoactivation slightly decreases, a behavior that can be attributed to difficulties in achieving homogeneous NP dispersion within the geopolymer matrix.

When comparing the 3 wt.% NP and 4 wt.% NP cases, no major differences are observed, with variations below 10%, which suggests, from a cost–benefit perspective, that increasing the NP content from 3 wt.% to 4 wt.% is not justified. At this stage of the analysis, no clear conclusions can be drawn regarding the influence of geopolymer compositional design (NaOH solution molarity or Na_2_SiO_3_:NaOH ratio) on the magnitude and trend of whiteness variation; however, preliminary indications suggest that a Na_2_SiO_3_:NaOH ratio of 2:1 favors higher increases in whiteness upon UV photoactivation.

**Figure 10:**
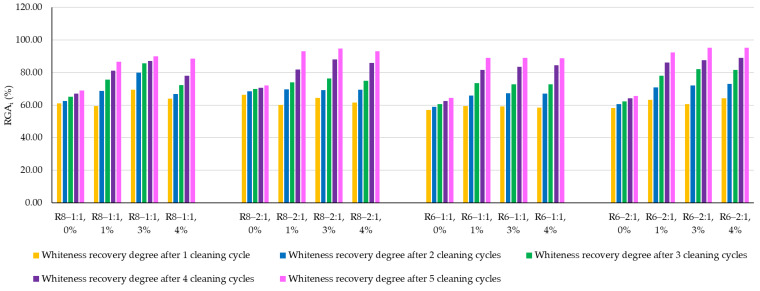
Whiteness recovery rate.

The whiteness recovery degree (RGA_i_), shown in Figure 10, mainly varies as a function of the number of washing–UV radiation–drying cycles. The more such cycles the material surface undergoes, the more the surface whiteness tends to return toward its initial values, due to the cumulative effect of oxidation and decomposition of the organic staining agent followed by its removal from the surface by washing water.

The experimental results tend to indicate that a higher Na_2_SiO_3_ content used in the preparation of the alkaline activator for fly ash, namely a Na_2_SiO_3_:NaOH ratio of 2:1, favors the discoloration of the Rhodamine B stain. This observation is supported both by the visual appearance of the samples (microscopy images after five cycles) and by the RGA_i_ indicator (where i is the number of cycles), which increases continuously with the number of cycles for all samples, but with a much steeper slope for specimens prepared with the alkaline activator having a Na_2_SiO_3_:NaOH ratio of 2:1.

In all cases, the recovery of the UV-activated surface appearance prior to staining is also positively influenced by an increase in the NP content available in the geopolymer matrix. After five washing–UV radiation–drying cycles, geopolymer compositions prepared with an alkaline activator characterized by a Na_2_SiO_3_:NaOH ratio of 2:1 achieve a recovery of the initial appearance exceeding 92%, whereas compositions prepared with an alkaline activator with a Na_2_SiO_3_:NaOH ratio of 1:1 recover 86–89% of the initial appearance recorded after photoactivation and before staining.

Regarding the influence of the NaOH solution molarity used to prepare the alkaline activator, no clear trend can be established; however, a slight favorable effect may be suggested for the 6 M concentration. This observation could be correlated with the influence of NaOH molarity on the porosity of the material. Thus, correlating the observations regarding the discoloration behavior of the Rhodamine B stain with the characteristics of the alkaline activator—and indirectly with the composite porosity—it can be stated that higher open porosity contributes to a better fixation of the staining agent on the material surface, but, on the other hand, during successive washing–UV radiation–drying cycles, it also facilitates its decomposition and removal, thereby enhancing the self-cleaning effect.

**Figure 11:**
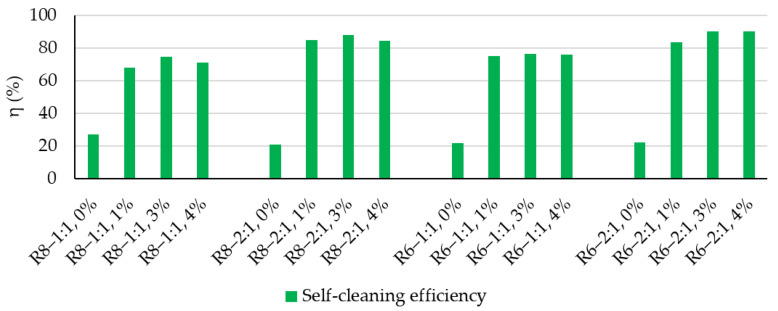
Self-cleaning efficiency indicator.

By evaluating the self-cleaning efficiency (η) after the completion of five cleaning cycles (Figure 11), it can be stated that the incorporation of TiO_2_ nanoparticles into the geopolymer matrix represents the key factor governing effective self-cleaning performance. However, identifying an optimal NP addition level is also crucial. In the present case, a 3 wt.% NP addition leads to higher efficiency values compared to 1 wt.% NT, which appears to be insufficient, or 4 wt.% NT, which is likely excessive for achieving good dispersion within the geopolymer matrix. At higher contents, local agglomeration may occur, inducing non-uniform self-cleaning capacity across the surface, with part of the NPs not being photoactivated and effectively acting as an inert additive.

Furthermore, as also indicated by the other quantification indicators, a Na_2_SiO_3_:NaOH ratio of 2:1 can be considered favorable for the development of good self-cleaning capacity, with efficiency values exceeding 83% for both geopolymer compositions prepared with alkaline activators based on 6 M NaOH solutions and those prepared with alkaline activators containing 8 M NaOH solutions.

The analysis of surface appearance (Table 3, Table 4, Table 5 and Table 6) supports the trends observed in the quantification indicators. As can be seen, after five photoactivation–washing–drying cycles, the Rhodamine B stain is largely discolored. However, for the R6-1:1 and R8-1:1 geopolymers, traces of the staining agent remain more visible and more uniformly distributed, maintaining a slight pink hue. In contrast, for the R6-2:1 and R8-2:1 geopolymer groups, the dye traces are barely perceptible and are unevenly localized, most likely because of more pronounced open porosity in the areas where residual dye persists.

The behavior of the reference samples—geopolymer mixtures without NP additions—in terms of self-cleaning capacity differs markedly from that of the NT-containing mixtures. It is clearly observed that UV exposure does not produce significant changes in surface whiteness, which increases by only 1–3 units (Figure 7). This slight increase is more likely attributable to possible ongoing geopolymerization reactions over time or to surface color non-uniformity, rather than to a genuine improvement in whiteness.

Upon staining with the Rhodamine B solution, similarly to the NT-containing geopolymer samples, a drastic reduction in whiteness is observed, with the surface becoming intensely colored with the characteristic pink hue. The values of the indicator characterizing this phenomenon (SGA_Rh_), shown in Figure 8, are close to those recorded for NT-containing composites, namely 35.4–45.7%.

As the photoactivation–washing–drying cycles proceed, the reference specimens exhibit a tendency toward stain discoloration, indicated by moderate increases in whiteness (Figure 7 and Figure 10) of 3–8 units from one cycle to the next; however, no clear general trend in the rate of whiteness recovery can be established. Moreover, the self-cleaning efficiency indicator (η) is significantly lower than that of the NT-containing samples, falling within the range of 21–27.3% after five photoactivation–washing–drying cycles.

Although much lower, the fact that the efficiency indicator (η) is not zero is attributed to the physical removal of staining agent particles by the washing water, combined, most likely, with partial degradation of the organic Rhodamine B compound, a process known to be induced by UV radiation exposure.

## 4. Conclusions

The aim of this work was to analyze the influence of TiO_2_ nanoparticle incorporation on the physico-mechanical characteristics and on the possibility of inducing smart, innovative self-cleaning behavior on the surface of geopolymer matrices prepared using locally sourced raw materials.

Based on the analysis of the relevant literature, a strong interest can be identified in the development of innovative technologies aimed at reducing the carbon footprint in the construction sector by limiting cement consumption, increasing the valorization of industrial by-products, and exploiting nanomaterials to enhance the performance of construction materials and to induce new, innovative functionalities, such as self-cleaning capacity. In this context, the development of alkali-activated fly ash-based geopolymers with self-cleaning capabilities induced by NP additions responds well to current technological and sustainability demands.

Based on the experimental results, the following conclusions can be drawn:

- The incorporation of nanoparticles into alkali-activated fly ash-based geopolymers leads to densification of the matrix, with the apparent density in the hardened state increasing continuously as the NP content increases from 1 wt.% to 4 wt.%. This parameter is influenced both by the compositional design of the geopolymer matrix—namely, the NaOH solution concentration used to prepare the alkaline activator and the Na_2_SiO_3_:NaOH ratio—and by the NP content. The use of an 8 M NaOH solution and a Na_2_SiO_3_:NaOH ratio of 2:1 reduces the magnitude of apparent density variation. The increase in apparent density relative to the reference mixture ranged from a minimum of 0.59% (R8-1:2, 1% NT) to a maximum of 3.74% (R6-1:1, 4% NT).
- The flexural tensile and compressive strengths are also influenced by the same three factors identified for apparent density: NaOH solution concentration, Na_2_SiO_3_:NaOH ratio, and NP addition. In general, an overall trend of improvement is observed up to a threshold level, beyond which further increases in NP content led to a reverse effect, namely, a reduction in mechanical strength. In the present experimental study, the optimal NP content was identified as 3 wt.%, for which the improvement in flexural tensile strength ranged from a minimum of 9.71% (R6-1:1, 3% NT) to a maximum of 24.32% (R6-2:1, 3% NT), while the improvement in compressive strength ranged from a minimum of 2.06% (R6-1:1, 3% NT) to a maximum of 6.98% (R8-2:1, 3% NT).
- Capillary water absorption, an indirect indicator of open porosity, is another parameter influenced by the compositional design of geopolymers with and without NP addition. In this case, the experimental results showed that the NaOH solution concentration, the Na_2_SiO_3_:NaOH ratio, and the NP content are all influencing factors, with 3 wt.% NPs providing the most significant benefits: a reduction in the capillary water absorption coefficient of up to 19.58% (R6-1:1, 3% NT), with reductions starting from 3.67% (R6-2:1, 1% NT).
- From the perspective of self-cleaning capacity, the experimental results indicated degradation of up to ~90% of the Rhodamine B dye after five complete cycles of UV radiation (10 h, 860 lux)–artificial rain washing (2 h)–drying (2 h, 70 °C)–dark storage (10 h, 23 ± 1 °C, 65 ± 5% relative humidity), indicating effective photocatalytic activity. In contrast, for the reference samples without NPs (0 wt.% NT), stain discoloration reached a maximum of only 27.3%. The discoloration observed for the reference composites is attributed mainly to the combined effect of physical removal of dye particles by the washing water and partial degradation of organic substances under UV radiation. The significant difference between the behavior of reference samples and NT-containing samples clearly demonstrates the induction of superior self-cleaning performance as a result of NP photoactivation.
- The lower self-cleaning activity, together with the deterioration of mechanical properties and increased water absorption observed for compositions containing 4 wt.% NT, is attributed to inhomogeneous nanoparticle distribution and particle agglomeration within the geopolymer matrix.

Overall, by analyzing the research hypotheses, it can be stated that the experimental results validate both the induction of changes in physico-mechanical characteristics and the imparting of self-cleaning functionality. The identified influencing factors are the amount of nanoparticles added to the binder matrix and the characteristics of the alkaline activator, as defined by the NaOH solution concentration and the Na_2_SiO_3_:NaOH ratio used in its preparation.

Considering all the aspects presented, two main directions for continuing the research can be identified, namely a more in-depth microstructural analysis correlated with the analysis performed at the macrostructural level, and the increase in the volume of experimental results by reducing the increment step of the NP addition, so as to obtain a dataset that allows statistical analysis together with the identification of the optimal amount of nanoparticle addition. Nevertheless, the value of the research derives, on the one hand, from demonstrating the possibility of obtaining an innovative material with a self-cleaning capability, which enables the sustainable and more environmentally friendly reuse of an industrial by-product. On the other hand, the study also contributes to enriching the information available in the specialized literature, including by indicating that approaching the research using a 1% increment step for the addition of NPs, although it reveals trends in the evolution of the composite characteristics and demonstrates the possibility of inducing self-cleaning capability, does not provide sufficient information for establishing an optimal composition, and therefore this increment step should be reduced.

The experimental results demonstrate the technical feasibility of using NPs in low proportions to develop alkali-activated geopolymer materials with advanced functional properties. The addition of semiconductor nanoparticles confers photocatalytic properties, opening the possibility of applying these materials in the production of prefabricated elements intended for pavements or wall cladding with a self-cleaning capability, for example, in buildings and public spaces.

## Figures and Tables

**Table 1:** Oxide composition of the fly ash used in the production of the geopolymer samples.

**Oxide** **composition (%)**	**SiO_2_**	**Al_2_O_3_**	**Fe_2_O_3_**	**CaO**	**MgO**	**SO_3_**	**Na_2_O**	**K_2_O**	**P_2_O_5_**
	46.94	23.83	10.08	10.72	2.63	0.45	0.62	1.65	0.25
	**TiO_2_**	**Cr_2_O_3_**	**Mn_2_O_3_**	**ZnO**	**SrO**	**CO_2_**	**P.C.**	**SiO_2_ + Al_2_O_3_**	**TiO_2_**
	0.92	0.02	0.06	0.02	0.03	-	2.11	70.77	

**Table 2:** Compositional characterization of geopolymer samples.

Code	NaOH Solution Concentration [M]	Na_2_SiO_3_/NaOH Ratio [Mass Ratio]	AA/FA Ratio [Mass Ratio]	NT Addition [% by Mass Relative to FA]
R6-1:1, 0% NT	6	1:1	0.92–0.95	0
R6-1:1, 1% NT				1
R6-1:1, 3% NT				3
R6-1:1, 4% NT				4
R6-2:1, 0% NT		2:1		0
R6-2:1, 1% NT				1
R6-2:1, 3% NT				3
R6-2:1, 4% NT				4
R8-1:1, 0% NT	8	1:1	0.92–0.95	0
R8-1:1, 1% NT				1
R8-1:1, 3% NT				3
R8-1:1, 4% NT				4
R8-2:1, 0% NT		2:1		0
R8-2:1, 1% NT				1
R8-2:1, 3% NT				3
R6-2:1, 4% NT				4

**Table 3:** Visual assessment of the surface appearance of R6-1:1 geopolymer composites.

R6-1:1					
	0% NPs	1% NPs	3% NPs	4% NPs	Magnification
Initial surface appearance	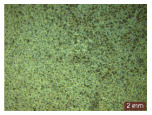	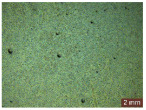	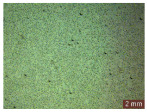	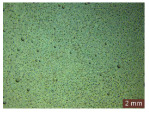	1×
	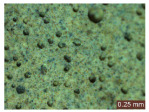	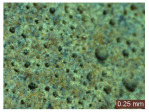	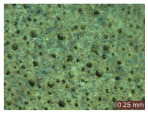	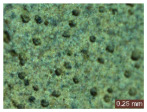	8×
Initial appearance after staining	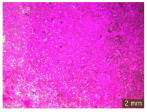	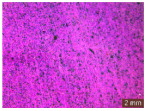	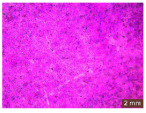	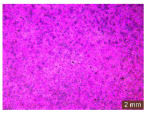	1×
	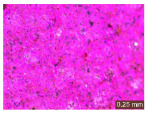	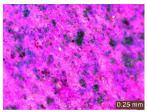	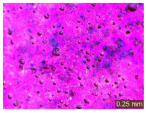	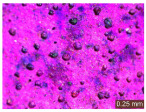	8×
Appearance after 5 cycles	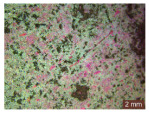	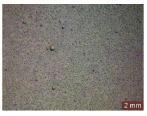	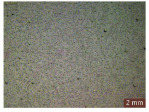	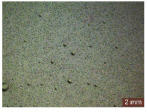	1×
	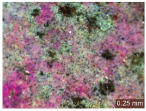	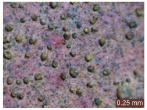	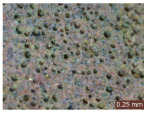	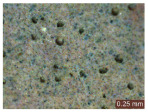	8×

**Table 4:** Visual assessment of the surface appearance of R6-2:1 geopolymer composites.

R6-2:1					
	0% NPs	1% NPs	3% NPs	4% NPs	Magnification
Initial surface appearance	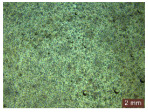	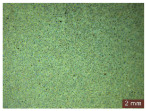	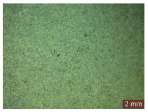	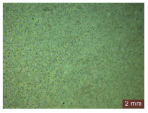	1×
	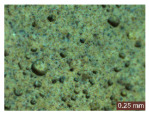	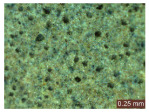	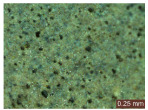	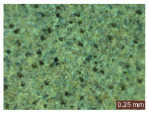	8×
Initial appearance after staining	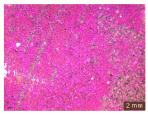	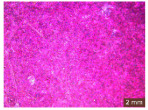	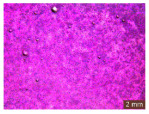	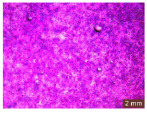	1×
	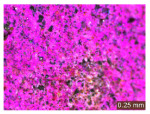	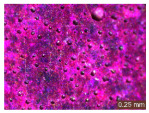	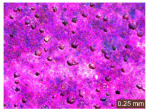	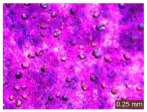	8×
Appearance after 5 cycles	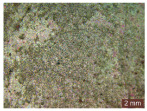	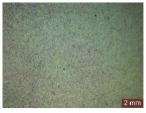	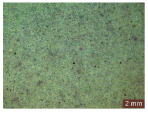	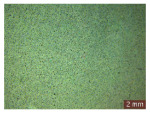	1×
	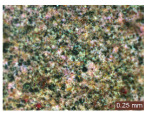	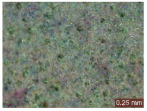	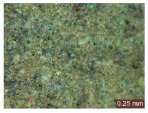	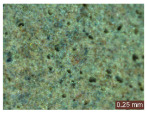	8×

**Table 5:** Visual assessment of the surface appearance of R8-1:1 geopolymer composites.

R8-1:1					
	0% NPs	1% NPs	3% NPs	4% NPs	Magnification
Initial surface appearance	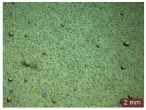	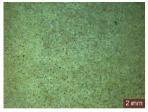	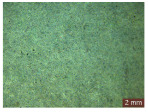	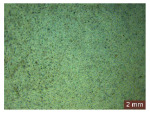	1×
	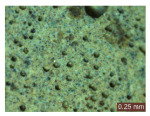	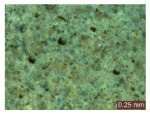	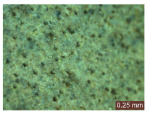	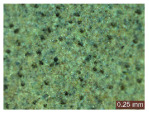	8×
Initial appearance after staining	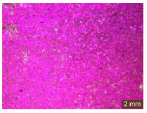	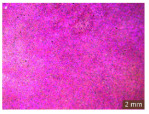	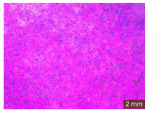	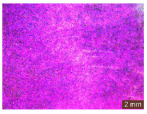	1×
	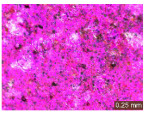	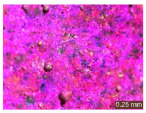	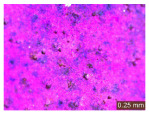	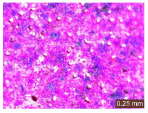	8×
Appearance after 5 cycles	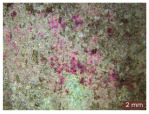	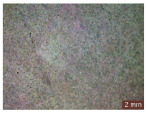	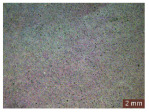	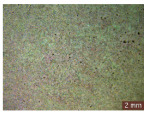	1×
	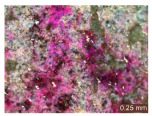	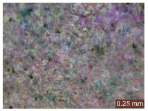	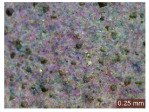	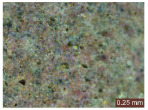	8×

**Table 6:** Visual assessment of the surface appearance of R8-2:1 geopolymer composites.

R8-2:1					
	0% NPs	1% NPs	3% NPs	4% NPs	Magnification
Initial surface appearance	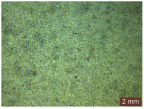	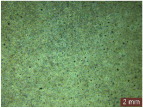	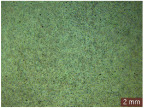	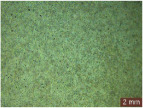	1×
	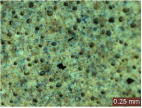	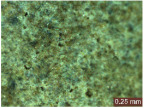	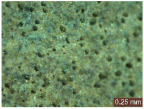	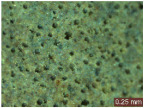	8×
Initial appearance after staining	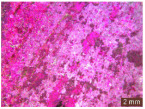	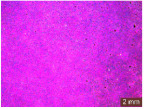	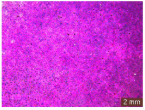	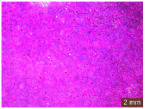	1×
	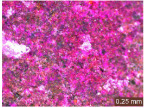	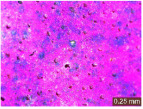	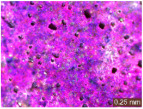	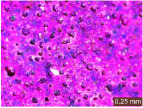	8×
Appearance after 5 cycles	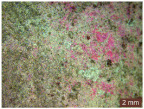	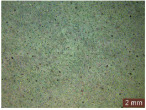	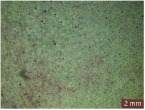	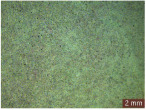	1×
	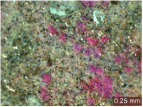	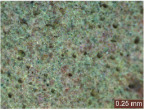	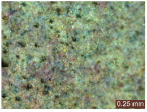	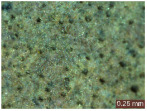	8×

## Data Availability

Data is contained within the article.
